# (*S*)-2-Amino-2-(2-chloro­phen­yl)cyclo­hexa­none

**DOI:** 10.1107/S1600536811009950

**Published:** 2011-03-19

**Authors:** Manfred Biermann, Kenneth I. Hardcastle, Nikolai V. Moskalev, Peter A. Crooks

**Affiliations:** a Resodyn Corporation, 130 North Main Street, Suite 600, Butte, MT 59701, USA; b Department of Chemistry, Emory University, Atlanta, GA 30322, USA; c Department of Pharmaceutical Sciences, College of Pharmacy, University of Kentucky, Lexington, KY 40536, USA

## Abstract

The crystal structure of the title compound, C_12_H_14_ClNO, was determined in order to confirm that the chiral center of the mol­ecule has an *S* configuration. The cyclo­hexa­none ring adopts a chair conformation. The 2-chloro­phenyl ring is slightly twisted from the axial C—N bond, with a N—C—C—C torsion angle of −5.7 (2)°. In the crystal, an inter­molecular N—H⋯O hydrogen bond links adjacent mol­ecules into an infinite chain, which propagates in the *b*-axis direction.

## Related literature

For background literature on the preparation and use of some anesthetics, see: Holtman *et al.* (2006 ▶); Heshmati *et al.* (2003 ▶); Kohrs & Durieux (1998 ▶). For information on the synthetic transformations used, see: Kolb *et al.* (1994 ▶); Parcell & Sanchez (1981 ▶); Senanayake *et al.* (1996 ▶); Yang & Davisson (1985 ▶).
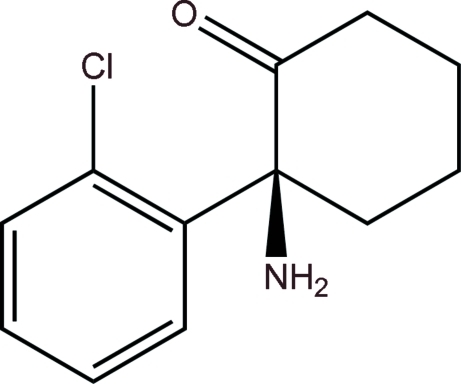


## Experimental

### 

#### Crystal data

C_12_H_14_ClNO
*M* *_r_* = 223.69
Orthorhombic, 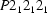

*a* = 7.2437 (5) Å
*b* = 7.4244 (5) Å
*c* = 20.4794 (15) Å
*V* = 1101.38 (13) Å^3^
*Z* = 4
Cu *K*α radiation
μ = 2.84 mm^−1^
*T* = 173 K
0.43 × 0.15 × 0.03 mm

#### Data collection

Bruker SMART APEX II diffractometer
Absorption correction: multi-scan (*SADABS*; Sheldrick, 1996 ▶) *T*_min_ = 0.375, *T*_max_ = 0.920
3449 measured reflections
1538 independent reflections
1521 reflections with *I* > 2σ(*I*)
*R*_int_ = 0.022

#### Refinement

*R*[*F* ^2^ > 2σ(*F* ^2^)] = 0.023
*wR*(*F* ^2^) = 0.059
*S* = 1.01
1538 reflections
136 parameters
H-atom parameters constrained
Δρ_max_ = 0.14 e Å^−3^
Δρ_min_ = −0.15 e Å^−3^
Absolute structure: Flack (1983 ▶), 545 Friedel pairs
Flack parameter: 0.060 (13)

### 

Data collection: *SMART* (Bruker, 2007 ▶); cell refinement: *SAINT* (Bruker, 2007 ▶); data reduction: *SAINT*; program(s) used to solve structure: *SHELXTL* (Sheldrick, 2008 ▶); program(s) used to refine structure: *SHELXTL*; molecular graphics: *SHELXTL*; software used to prepare material for publication: *SHELXTL* and *publCIF* (Westrip, 2010 ▶).

## Supplementary Material

Crystal structure: contains datablocks global, I. DOI: 10.1107/S1600536811009950/nk2080sup1.cif

Structure factors: contains datablocks I. DOI: 10.1107/S1600536811009950/nk2080Isup2.hkl

Additional supplementary materials:  crystallographic information; 3D view; checkCIF report

## Figures and Tables

**Table 1:** Hydrogen-bond geometry (Å, °)

*D*—H⋯*A*	*D*—H	H⋯*A*	*D*⋯*A*	*D*—H⋯*A*
N1—H1*A*⋯O1^i^	0.91	2.20	3.066 (2)	160

Symmetry code: (i) 
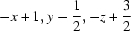
.

**Fig. 1.**
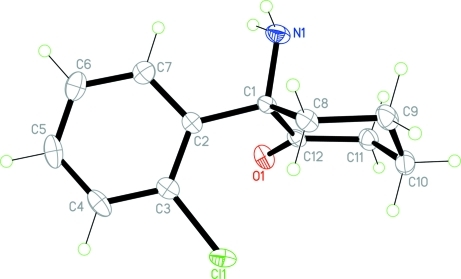
The asymmetric unit, with displacement ellipsoids drawn at the 30% probability level.

**Fig. 2.**
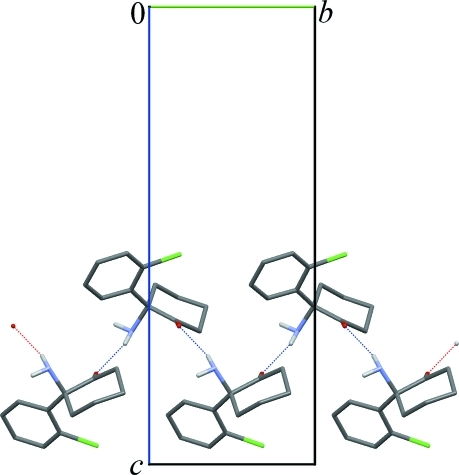
N–H···O hydrogen bonding interactions (blue dotted lines) in the crystal packing form an infinite chain.

## supplementary crystallographic information

### Comment

Ketalar^TM^, the racemic mixture of *R*- and *S*-Ketamines is becoming the sedative and anesthetic of choice for emergency sedation in children and victims with unknown medical history, *e.g.* from traffic accidents to battlefield conditions, because it causes minimal respiratory depression in comparison to other anesthetics (Heshmati *et al.*, 2003). *S*-Ketamine was found 3–4 times more potent as an anesthetic than its *R*-enantiomer, and twice as potent as Ketalar^TM^ with fewer side effects such as psychedelic, disorientation and anxiety (Kohrs & Durieux, 1998). *S*-Norketamine, the major metabolite of *S*-Ketamine in humans and animals, is emerging as a novel drug for treatment of neuropathic pain and for analgesia (Holtman *et al.*, 2006). To confirm the absolute configuration of (+)-norketamine, herein we report on the X-ray crystallographic characterization of crystalline *S*-norketamine.

The chirality of the molecule is confirmed (Figure 1). In the structure, the cyclohexanone ring adopts a chair conformation. The 2-chlorophenyl ring is slightly twisted from the axial C—N bond, with a torsion angle of -5.7 (2)°. In the crystal, an N–H···O hydrogen bond links adjacent molecules into an infinite chain which propagates in the *b*-axis direction (Figure 2).

### Experimental

With 2-chlorophenyl-1-cyclohexene as pro-chiral starting material, the enantioselective synthesis of *S*-norketamine was first time accomplished *via* a 3-step synthesis route. In the first step the chiral quarternary C-1 atom of the ketamine parent structure was generated in utilizing an adapted Sharpless-Asymmetric Dihydroxylation method (Kolb *et al.*, 1994). Asymmetric dihydroxylation was conducted with osmiumtetroxide modified with hydroquinine 1,4-phthalazinediyl diether ((DHQ)2PHAL) as chiral ligand in *tert*-butanol yielding (-)-(1*S*, 2*S*)-1-(2-chlorophenyl)cyclohexane-1,2-diol in 92% yield and with 82–85% ee after crystallization from *n*-heptane. In the second step (-)-(1*S*, 2*S*)-1-(2-chlorophenyl) cyclohexane-1,2-diol was subjected to the condition of the Ritter Reaction (Senanayake *et al.*, 1996) which produced (-)-(1*S*, 2*S*)-1-amino-1(2-chlorophenyl) cyclohexane-2-ol, which was obtained with 95% ee after crystallization from *n*-hexane. In the third step modified Jones Oxidation (Yang *et al.*, 1985) of (-)-(1*S*, 2*S*)-1-amino-1(2-chlorophenyl) cyclohexane-2-ol produced (*S*)-2-amino-2-(2-chlorophenyl)cyclohexanone ((+)-*S*- norketamine) which was initially obtained as a solid white crystalline material after crystallization from *n*-heptane (Mp. 68–69°C) which was previously described as an oil (Parcell & Sanchez, 1981). The chiral purity was ee 99% determined by chiral HPLC (Chiralpak AD—H column). The specific rotation of the free *S*-norketamine base was established to be [*a*]_D_ +3.2° (c = 2, EtOH). Intermediates and end product were characterized by infrared, NMR and MS-spectroscopy.

### Refinement

All non-hydrogen atoms were refined anisotropically. The hydrogen atoms were positioned geometrically and refined as riding atoms. The Flack parameter was determined from 545 Friedel pairs (Flack, 1983).

### Crystal data

**Table:**

C_12_H_14_ClNO	*F*(000) = 472
*M**_r_* = 223.69	*D*_x_ = 1.349 Mg m^−^^3^
Orthorhombic, *P*2_1_2_1_2_1_	Cu *K*α radiation, λ = 1.54178 Å
Hall symbol: P 2ac 2ab	Cell parameters from 3161 reflections
*a* = 7.2437 (5) Å	θ = 4.3–64.6°
*b* = 7.4244 (5) Å	µ = 2.84 mm^−^^1^
*c* = 20.4794 (15) Å	*T* = 173 K
*V* = 1101.38 (13) Å^3^	Block, colourless
*Z* = 4	0.43 × 0.15 × 0.03 mm

### Data collection

**Table:**

Bruker SMART APEX II diffractometer	1538 independent reflections
Radiation source: fine-focus sealed tube	1521 reflections with *I* > 2σ(*I*)
graphite	*R*_int_ = 0.022
ω scans	θ_max_ = 64.7°, θ_min_ = 4.3°
Absorption correction: multi-scan (*SADABS*; Sheldrick, 1996)	*h* = −7→7
*T*_min_ = 0.375, *T*_max_ = 0.920	*k* = −8→8
3449 measured reflections	*l* = −24→19

### Refinement

**Table:**

Refinement on *F*^2^	Secondary atom site location: difference Fourier map
Least-squares matrix: full	Hydrogen site location: inferred from neighbouring sites
*R*[*F*^2^ > 2σ(*F*^2^)] = 0.023	H-atom parameters constrained
*wR*(*F*^2^) = 0.059	*w* = 1/[σ^2^(*F*_o_^2^) + (0.0302*P*)^2^ + 0.1*P*] where *P* = (*F*_o_^2^ + 2*F*_c_^2^)/3
*S* = 1.01	(Δ/σ)_max_ < 0.001
1538 reflections	Δρ_max_ = 0.14 e Å^−^^3^
136 parameters	Δρ_min_ = −0.15 e Å^−^^3^
0 restraints	Absolute structure: Flack (1983), 545 Friedel pairs
Primary atom site location: structure-invariant direct methods	Flack parameter: 0.060 (13)

### Special details

**Table:**

Geometry. All e.s.d.'s (except the e.s.d. in the dihedral angle between two l.s. planes) are estimated using the full covariance matrix. The cell e.s.d.'s are taken into account individually in the estimation of e.s.d.'s in distances, angles and torsion angles; correlations between e.s.d.'s in cell parameters are only used when they are defined by crystal symmetry. An approximate (isotropic) treatment of cell e.s.d.'s is used for estimating e.s.d.'s involving l.s. planes.
Refinement. Refinement of *F*^2^ against ALL reflections. The weighted *R*-factor *wR* and goodness of fit *S* are based on *F*^2^, conventional *R*-factors *R* are based on *F*, with *F* set to zero for negative *F*^2^. The threshold expression of *F*^2^ > σ(*F*^2^) is used only for calculating *R*-factors(gt) *etc*. and is not relevant to the choice of reflections for refinement. *R*-factors based on *F*^2^ are statistically about twice as large as those based on *F*, and *R*- factors based on ALL data will be even larger.There were problems during data colleciton that were only realised after refinement of the results. The data were quite weak at high angle and although data were collected out to 0.85 Angstrons, the processed data were only 89% complete; however the overall statistics and quality of the results appeared quite good.

### Fractional atomic coordinates and isotropic or equivalent isotropic displacement parameters (Å^2^)

**Table:**

	*x*	*y*	*z*	*U*_iso_*/*U*_eq_	
C1	0.2928 (2)	0.4934 (2)	0.84577 (8)	0.0241 (3)	
C2	0.4469 (2)	0.3899 (2)	0.88115 (7)	0.0248 (4)	
C3	0.5543 (3)	0.4618 (2)	0.93085 (7)	0.0288 (4)	
C4	0.6938 (3)	0.3647 (2)	0.96124 (9)	0.0389 (4)	
H4	0.7637	0.4178	0.9954	0.047*	
C5	0.7303 (3)	0.1905 (3)	0.94148 (10)	0.0463 (5)	
H5	0.8264	0.1236	0.9617	0.056*	
C6	0.6268 (3)	0.1144 (2)	0.89243 (10)	0.0434 (5)	
H6	0.6508	−0.0055	0.8787	0.052*	
C7	0.4874 (3)	0.2131 (2)	0.86316 (8)	0.0336 (4)	
H7	0.4166	0.1583	0.8295	0.040*	
C8	0.1313 (2)	0.5361 (2)	0.89240 (8)	0.0301 (4)	
H8A	0.0653	0.4228	0.9026	0.036*	
H8B	0.1822	0.5838	0.9338	0.036*	
C9	−0.0066 (2)	0.6718 (2)	0.86506 (9)	0.0379 (4)	
H9A	−0.0712	0.6184	0.8271	0.045*	
H9B	−0.1001	0.7003	0.8988	0.045*	
C10	0.0903 (3)	0.8435 (2)	0.84417 (9)	0.0380 (4)	
H10A	0.1524	0.8986	0.8823	0.046*	
H10B	−0.0018	0.9306	0.8274	0.046*	
C11	0.2330 (3)	0.8039 (2)	0.79098 (8)	0.0340 (4)	
H11A	0.1691	0.7598	0.7514	0.041*	
H11B	0.2986	0.9165	0.7795	0.041*	
C12	0.3715 (2)	0.6647 (2)	0.81317 (7)	0.0258 (4)	
Cl1	0.52113 (6)	0.68279 (5)	0.958904 (19)	0.03641 (13)	
N1	0.2147 (2)	0.39391 (19)	0.79034 (7)	0.0358 (3)	
H1A	0.3079	0.3550	0.7641	0.054*	
H1B	0.1502	0.2974	0.8055	0.054*	
O1	0.53524 (16)	0.67977 (16)	0.80222 (6)	0.0351 (3)	

### Atomic displacement parameters (Å^2^)

**Table:**

	*U*^11^	*U*^22^	*U*^33^	*U*^12^	*U*^13^	*U*^23^
C1	0.0209 (9)	0.0303 (7)	0.0211 (8)	−0.0004 (7)	−0.0008 (7)	−0.0029 (6)
C2	0.0218 (9)	0.0330 (8)	0.0197 (8)	−0.0022 (6)	0.0041 (7)	0.0037 (6)
C3	0.0267 (10)	0.0373 (8)	0.0223 (8)	−0.0045 (7)	0.0038 (7)	0.0031 (6)
C4	0.0300 (10)	0.0599 (11)	0.0267 (9)	−0.0057 (8)	−0.0052 (8)	0.0135 (8)
C5	0.0389 (11)	0.0555 (11)	0.0444 (11)	0.0114 (10)	0.0014 (9)	0.0248 (9)
C6	0.0478 (13)	0.0346 (9)	0.0477 (12)	0.0070 (8)	0.0074 (10)	0.0117 (8)
C7	0.0382 (11)	0.0326 (7)	0.0300 (9)	−0.0014 (8)	0.0040 (8)	0.0029 (6)
C8	0.0232 (9)	0.0409 (9)	0.0262 (9)	−0.0019 (7)	0.0036 (7)	−0.0012 (7)
C9	0.0234 (9)	0.0546 (10)	0.0357 (9)	0.0059 (10)	0.0011 (7)	−0.0063 (7)
C10	0.0345 (10)	0.0427 (9)	0.0367 (10)	0.0112 (8)	−0.0040 (8)	−0.0027 (8)
C11	0.0351 (10)	0.0375 (8)	0.0293 (8)	0.0039 (8)	−0.0035 (8)	0.0031 (7)
C12	0.0279 (10)	0.0344 (8)	0.0150 (7)	0.0001 (7)	−0.0020 (6)	−0.0016 (6)
Cl1	0.0387 (2)	0.0428 (2)	0.0278 (2)	−0.00777 (19)	−0.00231 (17)	−0.00948 (14)
N1	0.0314 (9)	0.0453 (7)	0.0307 (8)	−0.0022 (7)	−0.0032 (7)	−0.0123 (6)
O1	0.0266 (7)	0.0477 (6)	0.0310 (6)	−0.0013 (6)	0.0023 (5)	0.0107 (5)

### Geometric parameters (Å, °)

**Table:**

C1—N1	1.468 (2)	C8—C9	1.525 (2)
C1—C2	1.537 (2)	C8—H8A	0.9900
C1—C8	1.543 (2)	C8—H8B	0.9900
C1—C12	1.545 (2)	C9—C10	1.517 (3)
C2—C3	1.388 (2)	C9—H9A	0.9900
C2—C7	1.394 (2)	C9—H9B	0.9900
C3—C4	1.389 (3)	C10—C11	1.530 (3)
C3—Cl1	1.7548 (16)	C10—H10A	0.9900
C4—C5	1.380 (3)	C10—H10B	0.9900
C4—H4	0.9500	C11—C12	1.511 (2)
C5—C6	1.375 (3)	C11—H11A	0.9900
C5—H5	0.9500	C11—H11B	0.9900
C6—C7	1.384 (3)	C12—O1	1.212 (2)
C6—H6	0.9500	N1—H1A	0.9100
C7—H7	0.9500	N1—H1B	0.9100
			
N1—C1—C2	113.13 (13)	C9—C8—H8B	108.8
N1—C1—C8	106.87 (14)	C1—C8—H8B	108.8
C2—C1—C8	111.20 (13)	H8A—C8—H8B	107.7
N1—C1—C12	102.84 (13)	C10—C9—C8	110.82 (15)
C2—C1—C12	110.31 (13)	C10—C9—H9A	109.5
C8—C1—C12	112.21 (13)	C8—C9—H9A	109.5
C3—C2—C7	115.98 (16)	C10—C9—H9B	109.5
C3—C2—C1	124.08 (15)	C8—C9—H9B	109.5
C7—C2—C1	119.93 (15)	H9A—C9—H9B	108.1
C2—C3—C4	122.46 (16)	C9—C10—C11	110.58 (15)
C2—C3—Cl1	121.54 (13)	C9—C10—H10A	109.5
C4—C3—Cl1	116.01 (14)	C11—C10—H10A	109.5
C5—C4—C3	119.62 (18)	C9—C10—H10B	109.5
C5—C4—H4	120.2	C11—C10—H10B	109.5
C3—C4—H4	120.2	H10A—C10—H10B	108.1
C6—C5—C4	119.67 (18)	C12—C11—C10	111.48 (14)
C6—C5—H5	120.2	C12—C11—H11A	109.3
C4—C5—H5	120.2	C10—C11—H11A	109.3
C5—C6—C7	119.77 (18)	C12—C11—H11B	109.3
C5—C6—H6	120.1	C10—C11—H11B	109.3
C7—C6—H6	120.1	H11A—C11—H11B	108.0
C6—C7—C2	122.49 (18)	O1—C12—C11	122.06 (16)
C6—C7—H7	118.8	O1—C12—C1	121.14 (16)
C2—C7—H7	118.8	C11—C12—C1	116.62 (15)
C9—C8—C1	113.91 (15)	C1—N1—H1A	109.3
C9—C8—H8A	108.8	C1—N1—H1B	109.2
C1—C8—H8A	108.8	H1A—N1—H1B	109.5
			
N1—C1—C2—C3	173.46 (15)	C1—C2—C7—C6	178.79 (17)
C8—C1—C2—C3	−66.3 (2)	N1—C1—C8—C9	−68.43 (18)
C12—C1—C2—C3	58.89 (19)	C2—C1—C8—C9	167.66 (14)
N1—C1—C2—C7	−5.7 (2)	C12—C1—C8—C9	43.57 (19)
C8—C1—C2—C7	114.58 (16)	C1—C8—C9—C10	−54.5 (2)
C12—C1—C2—C7	−120.26 (15)	C8—C9—C10—C11	60.3 (2)
C7—C2—C3—C4	−0.1 (2)	C9—C10—C11—C12	−56.4 (2)
C1—C2—C3—C4	−179.30 (15)	C10—C11—C12—O1	−137.39 (18)
C7—C2—C3—Cl1	179.79 (12)	C10—C11—C12—C1	47.45 (19)
C1—C2—C3—Cl1	0.6 (2)	N1—C1—C12—O1	−101.44 (18)
C2—C3—C4—C5	0.7 (3)	C2—C1—C12—O1	19.5 (2)
Cl1—C3—C4—C5	−179.21 (14)	C8—C1—C12—O1	144.07 (16)
C3—C4—C5—C6	−0.7 (3)	N1—C1—C12—C11	73.77 (17)
C4—C5—C6—C7	0.2 (3)	C2—C1—C12—C11	−165.31 (14)
C5—C6—C7—C2	0.4 (3)	C8—C1—C12—C11	−40.72 (19)
C3—C2—C7—C6	−0.4 (2)		

### Hydrogen-bond geometry (Å, °)

**Table:**

*D*—H···*A*	*D*—H	H···*A*	*D*···*A*	*D*—H···*A*
N1—H1A···O1^i^	0.91	2.20	3.066 (2)	160

Symmetry codes: (i) −*x*+1, *y*−1/2, −*z*+3/2.
